# The association between breast arterial calcification and atherosclerotic cardiovascular disease in an Australian population-based breast cancer case–control study

**DOI:** 10.1007/s11547-023-01611-y

**Published:** 2023-03-06

**Authors:** Sing Ching Lee, Sarah Pirikahu, Lin Fritschi, Terry Boyle, Carl Schultz, Elizabeth Wylie, Jennifer Stone

**Affiliations:** 1 Department of Cardiology, Royal Perth Hospital, Perth, Australia; 2 Medical School, University of Western Australia, Perth, Australia; 3 Genetic Epidemiology Group, School of Population and Global Health, University of Western Australia, Perth, Australia; 4 School of Population Health, Curtin University, Perth, Australia; 5 Australian Centre for Precision Health, Allied Health and Human Performance, University of South Australia, Adelaide, Australia; 6 BreastScreen Western Australia, Perth, Australia

**Keywords:** Atherosclerotic cardiovascular disease, ASCVD, Breast arterial calcification, BAC, Mammogram, Cardiovascular disease

## Abstract

**Purpose:**

Atherosclerotic cardiovascular disease (ASCVD) is a major cause of morbidity and mortality. Breast arterial calcification (BAC) on mammograms is not associated with breast cancer risk. However, there is increasing evidence supporting its association with cardiovascular disease (CVD). This study examines the association between BAC and ASCVD and their risk factors within an Australian population-based breast cancer study.

**Materials and methods:**

Data from the controls who participated in the breast cancer environment and employment study (BCEES) were linked with the Western Australian Department of Health Hospital Morbidity database and Mortality Registry to obtain ASCVD outcomes and related risk factor data. Mammograms from participants with no prior history of ASCVD were assessed for BAC by a radiologist. Cox proportional hazards regression was used to examine the association between BAC and later occurrence of an ASCVD event. Logistic regression was used to investigate the factors associated with BAC.

**Results:**

A total of 1020 women with a mean age of 60 (sd = 7.0 years) were included and BAC found in 184 (18.0%). Eighty (7.8%) of the 1020 participants developed ASCVD, with an average time to event of 6.2 years (sd = 4.6) from baseline. In univariate analysis, participants with BAC were more likely to have an ASCVD event (HR = 1.96 95% CI 1.29–2.99). However, after adjusting for other risk factors, this association attenuated (HR = 1.37 95% CI 0.88–2.14). Increasing age (OR = 1.15, 95% CI 1.12–1.19) and parity (*p*_LRT_ < 0.001) were associated with BAC.

**Conclusion:**

BAC is associated with increased ASCVD risk, but this is not independent of cardiovascular risk factors.

**Supplementary Information:**

The online version contains supplementary material available at 10.1007/s11547-023-01611-y.

## Introduction

Atherosclerotic cardiovascular disease (ASCVD), which includes myocardial infarction, stroke and peripheral artery disease, remains a major cause of death, and women are more likely than men to die from an ASCVD event [1, 2]. This disparity between women and men is partially due to women being more likely to have delayed presentations or diagnoses [3]. In addition, the underestimation of cardiovascular risk in women from standard screening methods contributes to under-prescription of preventative therapies [4]. A female-specific screening tool that simultaneously increases ASCVD awareness and reduces risk factors could reduce these inequalities, especially if it was widely available at a minimal cost.

**Fig. 1:**
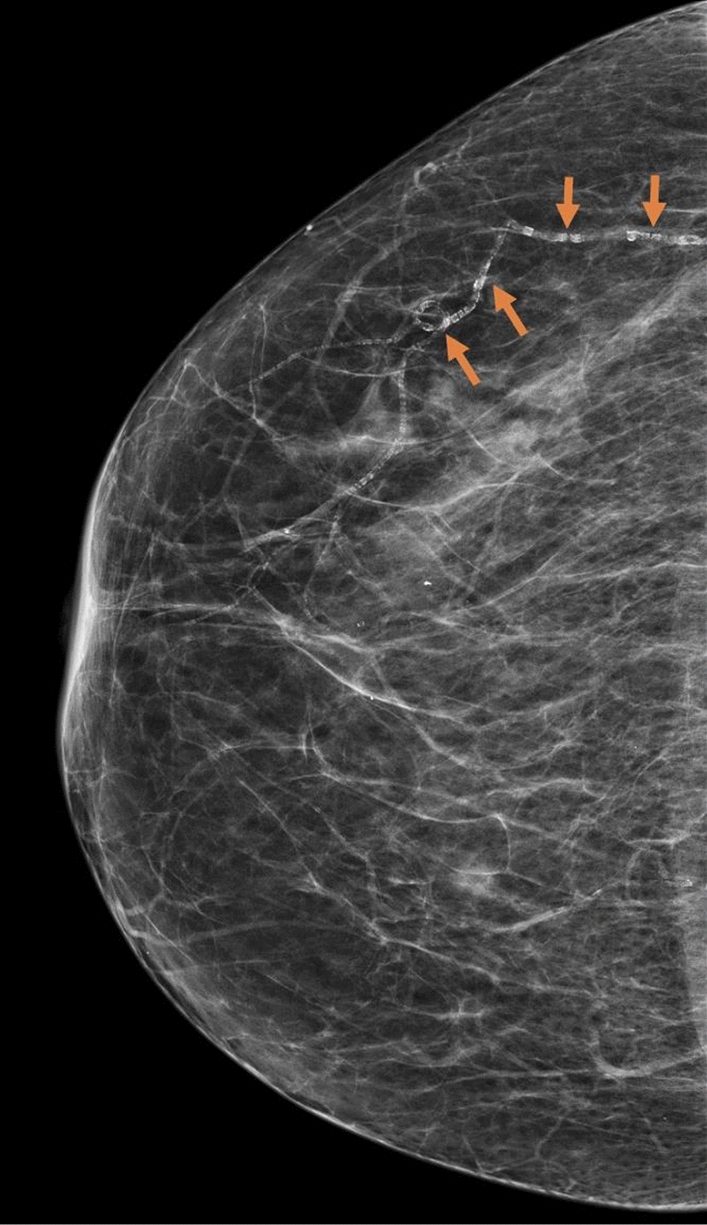
Breast arterial calcification on a mammogram (highlighted by the arrows)

Breast arterial calcification (BAC) is a common incidental finding in screening mammograms (Fig. 1). It is not known to be associated with breast cancer and is therefore, often not routinely mentioned in mammogram reports. A recent systematic review has shown that BAC may be a potential marker of coronary artery disease, diabetes mellitus and hypertension [5], which suggests that mammographic screening may help to identify women at increased risk of chronic diseases other than breast cancer. However, many of the previously published studies were cross sectional and did not specifically select a population of women with no prior history of ASCVD.

Utilizing data from an existing population-based breast cancer study, we selected a subset of women with no prior history of ASCVD to examine whether the presence of BAC was associated with the development of ASCVD. We also examined potential predictors of BAC, investigating if ASCVD risk factors were associated with the presence of BAC.

## Methods

### Study design and population

Control participants from a West Australian case–control study known as the Breast Cancer Environment and Employment Study (BCEES) were selected for this retrospective study [6]. In brief, controls were women aged between 18 and 80 years with an age distribution matched to the expected distribution of breast cancer in the population. Of the participating controls, those who had a mammogram between May 2009 and January 2011 were potentially eligible for this study.

Women with a prior history of ASCVD were excluded. These data were obtained through data linkage with BreastScreen Western Australia [7] and hospital morbidity and mortality databases from the Western Australian Department of Health Data Linkage Branch. ASCVD was defined as ischemic heart disease, stroke or peripheral artery disease, as described by Weng et al. [8], and the ICD-9 and -10 codes are detailed in Online Resource 1. Women were considered to have had an ASCVD event if they had a hospital admission for any of the relevant codes, or if they did not present to hospital but their cause of death was either one of the specified ICD codes, or was specified as cardiac arrest, myocardial infarction, cerebrovascular accident or a complication due to ischemic heart disease.

### Mammographic data

An expert radiologist (EW) blinded to ASCVD status visually assessed BAC as present or absent for each breast from medio-lateral oblique and cranio-caudal film screening mammograms. If BAC was present on either breast, a woman was classified as having BAC. Absolute dense area was obtained using the Cumulus software (Sunnybrook Health Sciences Centre, Toronto, Canada). Dense breast was defined as those whose absolute dense area was above the mean absolute dense area of the study population.

### Data collection of outcome and covariates

Incident ASCVD, as defined above, was ascertained through to the 11 October 2018, when follow-up ended, using the linkage to the hospital morbidity and mortality databases from the Western Australian Department of Health. Covariate data were obtained using the baseline epidemiological questionnaire data collected upon recruitment into the BCEES. This included information on age (years), body mass index (BMI, kg/m^2^), smoking (current, former and never), parity (number of births), breastfeeding (yes/no), hormone replacement therapy (HRT) use (yes/no) and contraceptive use (combined, other hormonal, unknown hormonal and no contraception). Other covariate data such as hypertension, dyslipidemia and diabetes mellitus were collected using data linkage. Hypertension, dyslipidemia and diabetes were defined as positive if a woman was ever diagnosed with ICD-9 and ICD-10 codes detailed in Online Resource 1.

### Statistical analysis

Descriptive statistics were used to characterize the study population. Univariable and multivariable logistic regression models were used to estimate odds ratios (OR) and 95% confidence intervals (CI) to describe the association between BAC and potential risk factors. The group with the greatest number of cases was selected as the reference group. Inclusion of covariates in the final multivariable model was determined using stepwise regression and a threshold of *p* = 0.05.

Hazards ratios (HR) and 95% CI were estimated using univariable and multivariable Cox proportional hazards model to describe the association between each factor and occurrence of a ASCVD event over time. Time to event was calculated in years from the date of screening mammogram until either the date of an ASCVD event, death (due to any cause) or the end of the study period. Women were right censored in the model if they died due to a cause other than ASCVD. The reference group was defined as the group with the greatest number of cases. Proportional hazard assumptions for the Cox models were assessed by Schoenfeld test and linearity of continuous variables via visualization of the Martingale residuals. Multivariable models were adjusted for age, BMI, hypertension, dyslipidemia, diabetes, smoking status, parity, breastfeeding, HRT use and contraceptive use. Inclusion of covariates in the final multivariable model was determined using stepwise regression and a threshold of *p* = 0.05. Factors considered important to ASCVD etiology (i.e., age, BMI) were retained in the model even if the *p* values were greater than the 0.05 level of significance. Interactions between BAC and hypertension or dyslipidemia were hypothesized *a-priori,* and evidence of interaction was assessed using likelihood ratio tests. Subset analyses were also carried out for women who had breast density measurements available.

## Results

**Fig. 2:**
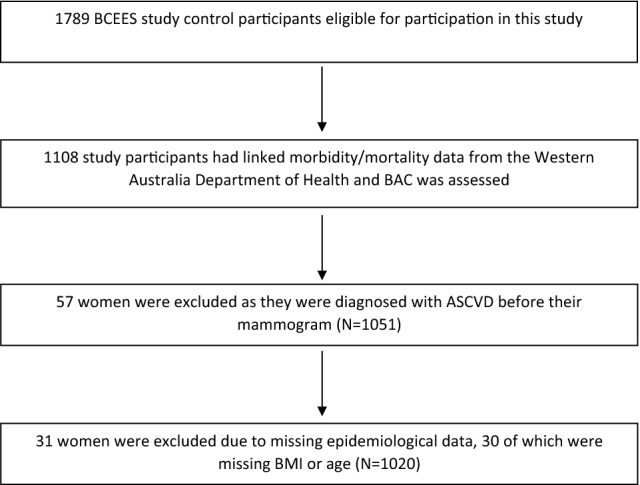
Flowchart of study subjects

Of the 1789 BCEES controls, 1108 had linked hospital morbidity, mortality data from the Western Australian Department of Health and had their mammogram assessed for BAC. Of these, 57 were diagnosed with ASCVD before their mammogram and were excluded. An additional 31 women were excluded due to incomplete epidemiological data. The final sample size of the dataset for analysis was 1020 (Fig. 2), and of these, 924 had mammographic breast density measures.

At the time of baseline assessment, the mean age was 60 (sd = 7.2 years). Overall, the prevalence of cardiovascular risk factors was low with only 1.8% having dyslipidemia and 3% having diabetes. Of the 1020 participants, BAC was found in 184 (18.0%) women. These women were more likely to be older (mean age: 65.1 vs. 59.3 years), have been diagnosed with hypertension (21.7 vs. 15.7%), diabetes (4.4 vs. 2.8%), never smoked (73.4 vs. 58.7%) and have previously breastfed (87.0 vs. 76.7%) (Table 1).

**Table 1:** Baseline cohort characteristics and associations between BAC and clinical and demographic factors

Risk factor		Total Mean ± SD or Count (%)	BAC (N = 184) Mean ± SD or Count (%)	No BAC (N = 836) Mean ± SD or Count (%)	Univariable analysis Odds ratio (95% CI)	Multivariable analysis Odds ratio (95% CI)
Age (years)		60.3 ± 7.0	65.1 ± 5.5	59.3 ± 6.9	1.15 (1.12–1.19)**	1.15 (1.12–1.19)**
BMI (kg/m^2^)		26.9 ± 5.3	26.3 ± 5.3	27.0 ± 5.3	0.97 (0.94–1.00)	0.97 (0.94–1.00)
Hypertension (vs. no)		171 (16.8)	40 (21.7)	131 (15.7)	1.49 (1.01–2.22)*	
Dyslipidemia (vs. no)		18 (1.8)	2 (1.1)	16 (1.9)	0.56 (0.13–2.47)	
Diabetes (vs. no)		31 (3.0)	8 (4.4)	23 (2.8)	1.61 (0.71–3.65)	
Smoker (vs. never)	Current	66 (6.5)	5 (2.7)	61 (7.3)	0.29 (0.12–0.76)*^a^	
	Former	328 (32.2)	44 (23.9)	284 (34.0)	0.56 (0.39–0.82)**	
Parity (vs. three +)	None	87 (8.5)	2 (1.1)	85 (10.2)	0.06 (0.02–0.27)**^b^	0.06 (0.01–0.25)**^c^
	One	71 (7.0)	5 (2.7)	66 (7.9)	0.21 (0.08–0.53)**	0.24 (0.09–0.62)**
	Two	407 (39.9)	56 (30.4)	351 (42.0)	0.44 (0.31–0.63)**	0.57 (0.39–0.82)**
Breastfed (yes)		219 (21.5)	24 (13.0)	195 (23.3)	0.49 (0.31–0.78)**	
HRT (vs. no)		566 (55.5)	111 (60.3)	455 (54.4)	1.27 (0.92–1.76)	
Contraception use (vs. no contraception)	Combined OC	474 (46.5)	87 (47.3)	387 (46.3)	0.98 (0.65–1.47)^d^	
	Other hormonal	180 (17.6)	31 (16.8)	149 (17.8)	0.90 (0.54–1.51)	
	Unknown	136 (13.3)	23 (12.5)	113 (13.5)	0.89 (0.51–1.55)	

*HRT* Hormone replacement therapy, *OC* Oral contraceptive, *CI* Confidence interval, *BAC* Breast arterial calcification; Significance is represented by * for *p* values < 0.05 and ** for *p* values < 0.01; for non-binary categorical variables, the like likelihood ratio test *p* values are provided ^a^ptrend = 0.0003; ^b^ptrend < 0.0001; ^c^ptrend < 0.0001; ^d^ptrend = 0.96

Univariable logistic regression (Table 1) showed that BAC was associated with increasing age (OR = 1.15 per year, 95% CI:1.12–1.19) and increased parity (OR = 0.06, 95% CI: 0.02–0.27, OR = 0.21, 95% CI: 0.08–0.53, OR = 0.44, 95% CI: 0.31–0.63 for none, one and two births, respectively, compared to three or more births). Women who had never breastfed were significantly less likely to have BAC (OR = 0.49, 95% CI: 0.31–0.78). Smoking was inversely associated with BAC (OR = 0.29, 95% CI: 0.12–0.76 and OR = 0.56, 95% CI: 0.39–0.82 for current and former smokers, respectively). In the multivariable analysis, only increasing age and parity showed evidence of association with BAC (Table 1).

A total of 80 women (7.8%) developed ASCVD with the majority (77.5%) having ischemic heart disease, and the average time to event (i.e., time between mammogram and ASCVD hospitalization or death) was 6.2 years (sd = 4.6 years). Table 2 shows the characteristics of women who did and did not develop ASCVD. Overall, the prevalence of cardiovascular risk factors was low, but, on average, women who developed ASCVD were older at baseline than those without ASCVD (64.2 vs. 60.0 years) and had a greater BMI (28.0 vs. 26.8 kg/m^2^). At baseline, ASCVD cases were more likely than those without ASCVD to have hypertension (55.0 vs. 13.5%) or dyslipidemia (8.8 vs. 1.2%) and were more likely to have taken hormone replacement therapy (70.0 vs. 54.3%).

**Table 2:** Univariable and multivariate risk of ASCVD in relation to clinical and demographic factors from Cox proportional hazards models

Risk factor		ASCVD (N = 80) Mean ± SD or Count (%)	No ASCVD (N = 940) Mean ± SD or Count (%)	Univariable analysis Hazard ratio (95% CI)	Multivariable analysis Hazard ratio (95% CI)
BAC (vs. no)		24 (30.0)	160 (17.0)	1.96 (1.29–2.99)**	1.37 (0.88–2.14)
Age (years)		64.2 ± 6.3	60.0 ± 7.0	1.09 (1.06–1.13)**	1.08 (1.04–1.11)**
BMI (kg/m^2^)		28.0 ± 6.6	26.8 ± 5.1	1.02 (0.98–1.05)	1.00 (0.97–1.04)
Hypertension (vs no)		44 (55.0)	127 (13.5)	4.15 (2.82–6.12)**	3.35 (2.23–5.04)**
Dyslipidemia (vs no)		7 (8.8)	11 (1.2)	3.85 (1.77–8.40)**	
Diabetes (vs. no)		8 (10.0)	23 (2.5)	2.66 (1.29–5.48)**	
Smoker (vs. never)	Current	5 (6.3)	61 (6.5)	1.43 (0.68–3.02)^a^	2.45 (1.15–5.22)*^b^
	Former	36 (45.0)	292 (31.1)	1.53 (1.02–2.28)*	1.83 (1.22–2.76)**
Parity (vs. three +)	None	9 (11.2)	78 (8.3)	1.04 (0.53–2.04)^c^	
	One	5 (6.3)	66 (7.0)	0.84 (0.38–1.85)	
	Two	26 (32.5)	381 (40.5)	0.80 (0.52–1.22)	
Breastfed (vs. yes)		23 (28.7)	196 (20.9)	1.32 (0.85–2.05)	
HRT (vs. no)		56 (70.0)	510 (54.3)	1.47 (0.99–2.18)	
Contraception use (vs. no contraception)	Combined OC	38 (47.5)	436 (46.4)	0.76 (0.48–1.19)^d^	
	Other hormonal	4 (5.0)	176 (18.7)	0.45 (0.23–0.90)*	
	Unknown	13 (16.2)	123 (13.1)	0.85 (0.46–1.57)	

*ASCVD* Atherosclerotic cardiovascular disease, *HRT* Hormone replacement therapy, *OC* Oral contraceptive, *CI* Confidence interval, *BAC* Breast arterial calcification; Significance is represented by * for *p* values < 0.05 and ** for *p* values < 0.01; for non-binary categorical variables, the like likelihood ratio test *p* values are provided ^a^ptrend = 0.11; ^b^ptrend = 0.005; ^c^ptrend = 0.73; ^d^ptrend = 0.12

In the univariable analysis (Table 2), women with BAC had 2.0 (95% CI: 1.29–2.99) times greater risk of developing ASCVD than those without BAC. As age increased, the risk of ASCVD increased by 1.1 times (95% CI: 1.06–1.13) each year. Hypertension, dyslipidemia or diabetes also resulted in significantly higher risk of ASCVD (HR = 4.15, 95% CI: 2.82–6.12, HR = 3.85, 95% CI: 1.77–8.40 and HR = 2.66, 95% CI: 1.29–5.48, respectively).

In the multivariable analysis, the association of BAC with ASCVD was no longer statistically significant (HR = 1.37, 95% CI: 0.88–2.14) after adjusting for age, BMI, hypertension and smoking. However, age, hypertension and smoking were significantly associated with risk of ASCVD. Dyslipidemia and diabetes were significantly associated with ASCVD risk in the univariate analysis but were omitted from the multivariable model due to collinearity with hypertension. Interactions between BAC and hypertension, smoking status and age were explored, but none were considered statistically significant (*p*_BACxhypertension_ = 0.09, *p*_BACxsmoker_ = 0.21 and *p*_BACxage_ = 0.29, respectively).

In our subset analysis of the 924 women who had breast density measurements available, the average breast density was 12.9 cm^2^ (sd = 14.6). Breast density was not associated with ASCVD or BAC (Table 3).

**Table 3:** Associations between breast density and ASCVD/BAC using Cox proportional hazards/logistic regression models for a subset (n = 924) of women

				Cox proportional hazards Outcome: ASCVD		Logistic regression Outcome: BAC	
		ASCVD (N = 71) Count (%)	No ASCVD (N = 853) Count (%)	Univariable Hazard ratio (95% CI)	Multivariable^†^ Hazard ratio (95% CI)	Univariable Odds ratio (95% CI)	Multivariable^‡^ Odds ratio (95% CI)
BAC	Yes	26 (33.8)	151 (17.7)	2.21 (1.43–3.40)**	1.48 (0.93–2.36)	NA	NA
	No	47 (66.2)	702 (82.3)	Reference	Reference	NA	NA
Dense breasts	Yes	25 (35.2)	311 (36.5)	0.86 (0.56–1.32)	1.05 (0.67–1.63)	0.86 (0.61–1.23)	1.21 (0.82–1.79)
	No	46 (64.8)	542 (64.8)	Reference	Reference	Reference	Reference

*BAC* Breast arterial calcification, *ASCVD* Atherosclerotic cardiovascular disease, *NA* Not applicable. Significance is represented by * for *p* values < 0.05 and ** for *p* values < 0.01

^†^Adjusted for BAC, age, BMI, hypertension, smoking status

^‡^Adjusted for age, BMI, parity

## Discussion

This is the first study to examine the associations of BAC with ASCVD risk factors and clinical events in an Australian population. Similar to previous studies conducted in other populations [9, 10], we found that BAC was present in 18% of a sample population of women attending a free mammographic screening program, BreastScreen WA. Contrary to previous reports, we found that BAC was not associated with the development of ASCVD events in women with no prior history of ASCVD. However, consistent with a recent meta-analysis [11], we report positive associations between BAC and age and parity.

### BAC and its risk factors

Similar to previous studies [12–15], BAC was associated with age in our study. In contrast to previous cohort studies [10, 13, 16, 17] including a recent systematic review [5], we found no associations of BAC with hypertension or diabetes. However, in studies with relatively healthier populations, no associations between BAC with diabetes, hypertension and dyslipidemia were found [18–20]. This supports our findings as our population were relatively healthy as evidenced by the low prevalence of dyslipidemia (2%) and diabetes (3%).

We report a positive association between BAC and parity. Reproductive factors, including parity [11, 13, 17, 18, 21–24], have previously been shown to be associated with BAC. A potential mechanism by which parity may induce BAC formation could be the transient increase in calcium required for breastfeeding and fetal growth [22, 25]. We found no association of BAC with HRT use and contraceptive use although a history of HRT use [11, 14, 16, 22, 26, 27] and oral contraceptive use [14, 24] was inversely associated with BAC formation in some studies. This suggests that estrogen may not have a role in the formation of BAC. However, studies that have examined HRT and risk of CVD suggest that other factors, which are not accounted for in our analysis, may be important. These include the formulation of HRT and duration of HRT use as well as when HRT was started relative to the age of onset of menopause. These factors need to be explored in greater detail to understand if estrogen has a role in BAC formation.

### BAC and ASCVD events

We report that the association found between BAC and ASCVD, in a population of women without prior ASCVD events, is not independent of age and other risk factors. This finding is in contrast to the first study that reported a 2.11-fold increased risk of ASCVD in subjects with BAC [28]. However, that cross-sectional study included 865 women with several cardiovascular risk factors (27% had dyslipidemia, 30% had hypertension, 26% were smokers, 6% had diabetes, 38% had a family history of ASCVD) and a high prevalence of ASCVD (18% of their study population).

More recently, a large prospective cohort study of 5059 women with no prior history of ASCVD found that BAC was associated with a 1.51-fold increased risk of developing ASCVD after a mean follow-up of 6.5 years [17]. Notably, a large proportion of their population at baseline was already on cholesterol lowering drugs (73%) or had hypertension (20%) or diabetes (13%). Similarly, in another recent very large prospective study of 57,867 women who attended screening mammogram in Sweden from January 2011 to March 2013, the presence of BAC was also found to be associated with a significant 5-year absolute risk of developing ASCVD (HR 1.09 (95% CI 1.05–1.13) and another cardiovascular risk factor. Those with BAC were also 1.5 times more likely to die from ASCVD. However, in the subset of women with no prior history of ASCVD or cardiovascular risk factors (*n* = 47,023), these associations were no longer seen with the exception of incident hypertension. These results support our study’s findings as the majority of our study population were relatively healthy, with a low prevalence of dyslipidemia and diabetes. Taken together, these data suggest that BAC may not be a good marker of future ASCVD events in healthy women. However, BAC may have utility as a marker of future cardiovascular risk in women with established cardiometabolic disease.

BAC severity, which was not assessed in our study, may have a role in the association of BAC and ASCVD. A systematic review has shown a quantitative association between BAC severity, assessed using subjective methods, and coronary artery disease [29]. A subsequent study using novel techniques to objectively measure BAC showed an association between the presence of BAC and ASCVD but no dose–response relationship [17]. A guideline on quantifying and reporting of BAC severity would be useful in future studies to investigate whether BAC severity is a factor in developing ASCVD. In addition, it is important to have long follow-up periods in order to detect a significant association between BAC and the development of ASCVD. In our study of a healthy population who are at low risk of future clinical events, the follow-up may not have been long enough.

### Breast density, ASCVD and BAC

In our cohort, breast density was not associated with ASCVD or BAC. Interestingly, two prior studies found that women with less dense breasts have a higher risk of CVD [29, 30]. More specifically, among those with no prior history of ASCVD or cardiovascular risk factors (*n* = 47,023), Grassmann et al. [30] found that dense breast was inversely associated with hypertension, peripheral vascular disease and diabetes mellitus. More studies are needed to assess this association. To our knowledge, we are the first to report that there is no association between breast density and BAC. The relevance of less dense breasts to CVD this remains to be determined but supports the idea that BAC and breast density represent two separate pathophysiological effects.

## Limitations

As our ASCVD events are determined primarily from data linkage and only events involving hospitalization were reported, it is possible that we have underestimated the number of ASCVD events or prevalence of cardiovascular risk factors, especially the presence of diabetes mellitus as we only had data on the number of newly diagnosed diabetes mellitus cases and not people with pre-existing diabetes mellitus. There is also a possibility that we have not excluded all women with a history of ASCVD or included all women who developed ASCVD, as they may not have attended a hospital in Western Australia. There are also other potential confounding factors, such as renal disease and statin use, that could not be accounted for in our study as these data were not available. Alternatively, the follow-up duration may not be sufficient to observe an association between BAC and ASCVD.

The quantification of BAC may provide further insight into the association of BAC and ASCVD, but this was not explored in our study.

## Conclusion

BAC is a marker of increased ASCVD risk, but this is not independent of cardiovascular risk factors. Whether BAC may be used to detect untreated ASCVD risk factors needs further study. The reporting of BAC on routine mammogram will assist in assessing the clinical utility of mammograms to screen for ASCVD.

## Supplementary Information

Below is the link to the electronic supplementary material.

Supplementary file1 (DOCX 15 kb)
