# Supplementary material for: The association between breast arterial calcification and atherosclerotic cardiovascular disease in an Australian population-based breast cancer case–control study
Source: Radiol Med. 2023 Mar 6;128(4):426–33. doi: 10.1007/s11547-023-01611-y (PMC10119204; doi:10.1007/s11547-023-01611-y)
Supplement: Supplementary file 1 — Supplementary file1 (DOCX 15 kb) [file 11547_2023_1611_MOESM1_ESM.docx]

**The association between breast arterial calcification, atherosclerotic cardiovascular disease and its risk factors within a breast cancer case-control study**

Sing Ching Lee^1,2^*†, Sarah Pirikahu^3^*, Lin Fritschi^4^, Terry Boyle^5^, Carl Schultz^1,2^, Liz Wylie^2,6^, Jennifer Stone^3^

^1^ Department of Cardiology, Royal Perth Hospital

^2^ Medical School, University of Western Australia

^3^ Genetic Epidemiology Group, School of Population and Global Health, University of Western Australia

^4^ School of Population Health, Curtin University, Perth

^5^ Australian Centre for Precision Health, Allied Health and Human Performance, University of South Australia, Adelaide

^6^BreastScreen Western Australia

*Joint first authors

†Corresponding author: [sing.lee@research.uwa.edu.au](mailto:sing.lee@research.uwa.edu.au) (ORCID ID: 0000-0003-0874-8796)

Word count: 2641

**Online Resource 1: ICD-9 and ICD-10 codes used to identify atherosclerotic cardiovascular disease cases and its risk factors**

|  | ICD-9CM | ICD-10CM |
| --- | --- | --- |
| Ischaemic heart disease | 410-414 (excludes 414.1) | I20-I25 (excludes I25.3, I25.4) |
| Transient ischaemic attacks | 435 | G45 |
| Stroke | 433, 434, 436, 437.0, 437.1 | I63, I65, I66, I67.2, I67.81, I67.82, I67.83, I67.84 |
| Peripheral artery disease | 440, 443.8, 443.9, 444, 445 | I70, I73.9, I74, I75 |
| Procedures related to ASCVD | V45.81, V45.82 | Z98.6, Z95.1, Z95.5, Z95.82, Z95.9 |
| Hypertension | 401-405 | I10, I11, I12, I13, I15 |
| Dyslipidaemia | 272 | E78 |
| Diabetes | 250 | E10, E11 |

Adapted from: Weng W, Tian Y, Kong SX, et al (2019) The prevalence of cardiovascular disease and antidiabetes treatment characteristics among a large type 2 diabetes population in the United States. Endocrinology, Diabetes & Metabolism 2:e00076. https://doi.org/10.1002/edm2.76
